# Stable and unstable regions of the Lorenz system

**DOI:** 10.1038/s41598-018-33010-z

**Published:** 2018-10-08

**Authors:** Bing Lu Shen, MingHao Wang, PengCheng Yan, HaiPeng Yu, Jian Song, Chao Jiu Da

**Affiliations:** 1 School of Atmospheric Sciences, Chengdu University of Information Technology, Chengdu, Sichuan China; 2 School of Mathematics and Computer Science Institute, Northwest Minzu University, Lanzhou, Gansu China; 3 School of Changwang, Nanjing University of Information Science and Technology, Nanjing, Jiangsu China; 4 Institute of Arid Meteorology, CMA, Lanzhou, Gansu China; 5 College of Science, Inner Mongolia University of Technology, Hohhot, Inner Mongolia China

## Abstract

The stable and unstable regions of the Lorenz system are studied. We discuss the relationship between the initial conditions and both these regions, specifically, the preference for the trajectory of the Lorenz system to move towards the left or right equilibrium-point region from an initial point and the residence time of a trajectory in an equilibrium-point region. For this purpose, the four-rank Runge–Kutta algorithms and mathematical derivations are used, whereas a statistical method for the residence times is used. We conclude that the stable and unstable regions are intrinsic to the Lorenz system and have no correlation with the initial conditions; indeed, these regions do not change given different initial conditions. The trajectory of the Lorenz system tends towards the left equilibrium-point region locally, with an average residence time of 8.74 but only 5.789 for the right equilibrium-point region. In general, the system prefers the right equilibrium-point region for which the jump frequency of trajectories to the right region is 535 but only 465 to the left region from the initial conditions for the first time.

## Introduction

In 1963, Lorenz analysed the nonlinear effects of convection using an equation that modelled the atmosphere, which is the Lorenz equation. Using a simplified mathematical version of this model, he found that when its parameters had particular values, the trajectory of the convective system becomes complex and uncertain, leading to unpredictable behaviour in the trajectories^1^. Based on the Lorenz equation, different applications and solving solution method are proposed^2–4^. This unpredictable behaviour originates from the instability of the Lorenz equation, due to the atmosphere abrupt change.

Around 1953, Hadamard constructed a counterexample showing that a differential equation was sensitive to the initial conditions, i.e., the initial values of the variables^5^. This counterexample has the same meaning as the unpredictable behaviour of the Lorenz equation, or the atmosphere abrupt change. In the mid-twentieth century, Thom studied the abrupt changes using differential operators and obtained several conclusions, which formed the mathematical basis for catastrophe theory^6^. Subsequently, Zeeman extended the notion of abrupt changes to include a wide range of applications^7^. The Lorenz equation played a role in confirming Hadamard’s counterexample concerning numerical experiments, finding examples for the catastrophe theory of Thom and Zeeman, and verifying the phenomenon associated with abrupt changes in climate.

From atmospheric observation data, discontinuities and jumps in meteorological variables are found, corresponding to abrupt changes in the atmosphere. These studies were begun in the 1930s, and thoroughly illustrated the point that the motion of the atmosphere undergoes abrupt changes both in time and in space^8–13^. The nature of this phenomenon indicates that the atmospheric system is a chaotic system^14^. The definition of the abrupt change is summarized and provided method to detect it^15,16^ but refer mainly to the classification of abrupt changes in the climate system. In regard to time-series periodic characteristics, theoretical study of abrupt changes is also taken^17^. With abundant meteorological observation data, various scholars researched the theme of abrupt changes in regional climate systems and the predictive trends in their evolution, the mechanism of which was also discovered^18–27^. With the application of mathematical physics techniques, many detection methods have been proposed, put into practice, and proved effective^28–32^. From palaeogeography, abrupt changes in climate have also had certain influences on the rise and decline of ancient civilizations^33,34^. Starting with the Lorenz equations, the dynamics of weather transition periods was studied in regard to numerical weather predictions. Through an analysis of the stability of equilibrium points of the Lorenz equations, surfaces that demarcated the stable and unstable regions were obtained, and new methods and theory applicable to the detection of abrupt changes in climate were obtained^4^.

As the atmospheric dynamical equations are nonlinear, a solution that is unstable can be interpreted as having global stability and local instability. Global stability means that no matter what the initial state is, the solution curve eventually shrinks to a stable attractor in the phase space. In contrast, local instability implies that the curve jumps irregularly between different tracks. Concerning this later feature, we analyse the microscopic features of the curve in different equilibrium regions to be able to predict if an abrupt change in the system is about to occur.

## Theoretical foundation

The Lorenz system is the set of nonlinear equations

1 $$\{\begin{array}{c}\frac{dx}{dt}=10(-x+y)\\ \frac{dy}{dt}=28x-y-xz\\ \frac{dz}{dt}=xy-\frac{8}{3}z\end{array}.$$

**Figure 1:**
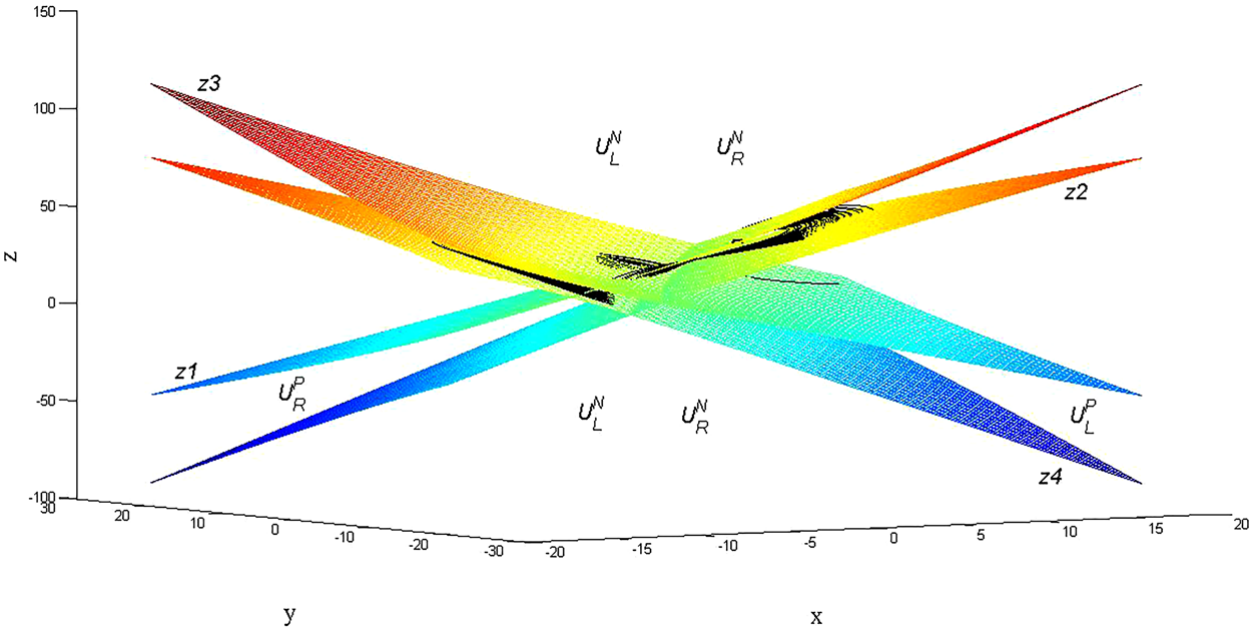
Nereus and Proteus regions of the Lorenz equation^4^.

Two equilibrium points, labelled left and right, exist for this system; they are points $L(-6\sqrt{2},-6\sqrt{2},27)$ and $R(6\sqrt{2},6\sqrt{2},27)$. The boundary surfaces of the stable and unstable regions, named Nereus and Proteus, have been obtained^4^, allowing for a visualization of these regions (Fig. 1)^4^. From the conclusions and methods presented in [4], we shall investigate three topics:

1. The relationship between the Nereus/Proteus region and the initial conditions,
2. The preference of trajectories for the left/right equilibrium point area,
3. The residence time of trajectories in the equilibrium point area

## Experiment

### Relationship between the Nereus/Proteus region and the initial conditions

Consider the following initial conditions of the Lorenz system, equation (1): (8, −19, 30), (15, −14, 78), (14, −5, 90), (−12, 1, 80), (−5, 17, 20), (−19, 10, −20), (−16, 11, −40), (−11, −1, −50), (15, 0, −55), (6, −19, −20). Using the four-rank Runge–Kutta algorithms, an incremental step of 0.01 over the integral interval [0, 10], and a truncation error is 0.01^3^, the surfaces z1 and z2 were found to have an analytic expression of the form^4^

2a $${F}_{1}(x,y,z)=-13.8546{(0.8450x-0.4380y-0.3322z+5.5159)}^{2}+0.0940(-0.3313x+0.1718y-0.8529z+24.3817{)}^{2}+0.0940{(0.3650x+1.0534y-0.0880z-9.6587)}^{2}.$$

Similarly, the surfaces z3 and z4 have an analytic expression of the form^4^

2b $${F}_{2}(x,y,z)=-13.8546{(0.8450x-0.4380y+0.3322z-5.5159)}^{2}+0.0940(-0.3313x+0.1718y+0.8529z-24.3817{)}^{2}+0.0940{(0.3650x+1.0534y+0.0880z+9.6587)}^{2}.$$

**Figure 2:**
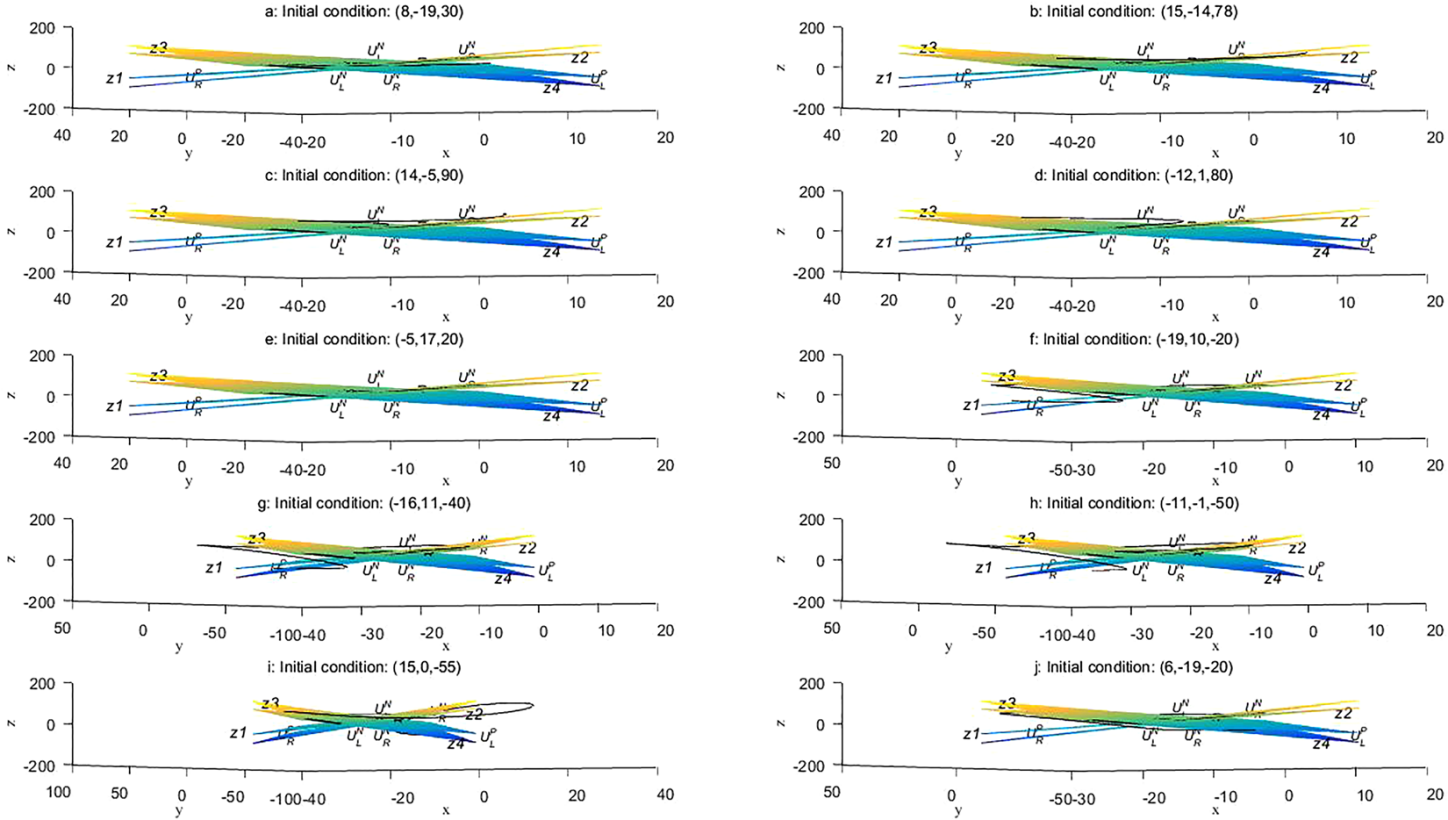
Lorenz trajectories for various initial conditions: (**a**) (8, −19, 30); (**b**) (15, −14, 78); (**c**) (14, −5, 90); (**d**) (−12, 1, 80); (**e**) (−5, 17, 20); (**f**) (−19, 10, −20); (**g**) (−16, 11, −40); (**h**) (−11, −1, −50); (**i**) (15, 0, −55); (**j**) (6, −19, −20).

Numerical experiments were then performed, the results of which are presented in Fig. 2(a–j).

From an analysis of these numerical results, we found that for different initial conditions, the Nereus and Proteus regions remain unchanged, which implies that these areas are inherent to the Lorenz system, equation (1), and are independent of the initial conditions for the point of the numerical test. Functions *F*_1_(*x*, *y*, *z*) and *F*_2_(*x*, *y*, *z*) are obtained from the Lorenz system^4^, and because of this, it is clear that surfaces z1, z2, z3, and z4 are independent of the initial conditions. Therefore, the Nereus and Proteus regions are unrelated to the initial field. We conclude that the Nereus and Proteus regions are intrinsic to the Lorenz system from theoretical considerations and numerical experiments. The atmospheric flow system subject to simplifications can be modelled by the Lorenz system, and hence we have much reason to believe that the atmospheric system has its own Nereus and Proteus regions. Similarly, these areas are intrinsic to the atmospheric flow system regardless of the values of the initial conditions. The Nereus and Proteus regions of the Lorenz system, which is a time-invariant system, are independent of the time variable as well. The atmospheric flow system is a general multi-equilibrium system written as

3 $$\{\begin{array}{c}\frac{d{x}_{1}}{dt}={f}_{1}({x}_{1},{x}_{2},\ldots ,{x}_{n},t)\\ \frac{d{x}_{2}}{dt}={f}_{2}({x}_{1},{x}_{2},\ldots ,{x}_{n},t)\\ \ldots \ldots \\ \frac{d{x}_{n}}{dt}={f}_{n}({x}_{1},{x}_{2},\ldots ,{x}_{n},t)\end{array}$$

Being a nonautonomous system, the operators *f*_*i*_(*i* = 1, 2, …, *n*) have an independent variable *t*, and the Nereus and Proteus regions are identified by these operators^4^; therefore, they are proportional to time *t*. Moreover, it is easy to show that the Nereus and Proteus regions of the atmospheric system vary over time. This may explain why some predictors of weather forecasts change over time. Predictions for past time periods are good but are not so good in true forecasts. Multiple-point equilibrium systems are also common in art designs. The Logo of the Pittsburgh Zoo is visually a two-point equilibrium system (see the following link: http://www.pittsburghzoo.org/).

### Preference of the trajectory

**Figure 3:**
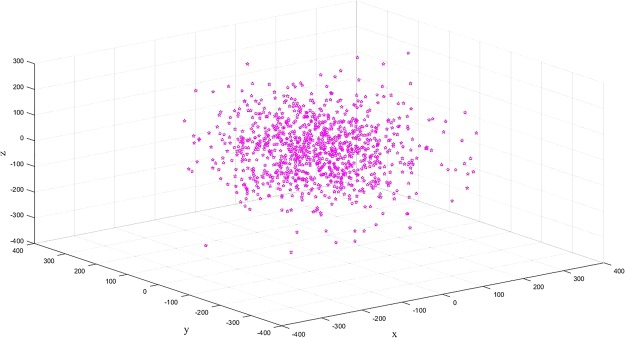
Scatter diagram of the initial jump.

A dynamical system can have a global stability, which means no matter what the initial conditions are, the trajectory will converge to an attractor. Local instability occurs when a system jumps between different trajectories (as well as regions with different equilibrium points). The attractor of the dynamical system forms from the interaction between global stability and local instability. The attractor of the Lorenz system, equation (1), is the famous *butterfly wings*, which has a left and a right branch. Nevertheless, for the two regions surrounding these two equilibrium points, is there a bias in the evolution trajectory of the system towards one or the other region? To answer this question, we performed the following numerical experiments. Using a PC, we selected randomly 1000 initial points:(*x*_*i*_, *y*_*i*_, *z*_*i*_) *i* = 1, 2, 3, …, 1000, for which the magnitude is of order 10^2^. We registered from which of the two regions (left or right) the trajectory made the first jump; Fig. 3 gives the scatter diagram of this initial jump.

**Figure 4:**
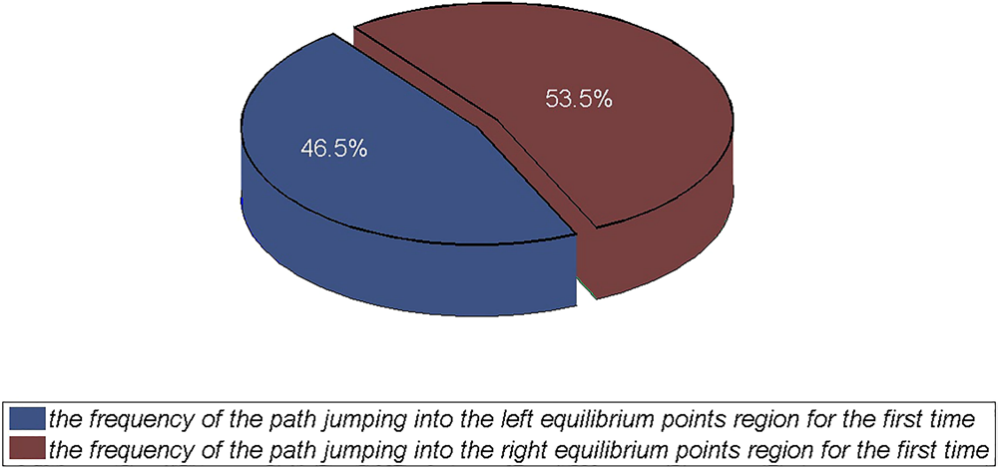
Frequency comparison chart of the path of the Lorenz system to the left or right equilibrium point region.

We find that the jump frequency of the trajectories to the left region is 465, whereas that to the right is 535 (Fig. 4). We conclude that in general the trajectory slightly favours the right equilibrium-point region when the trajectory converges to the attractor. For the atmospheric flow system, there are several equilibrium points. This system may similarly favour some of these equilibrium points and by-pass others. Trajectory jumps into this latter type of equilibrium point may correspond to extreme events^35^.

### Residence time of the trajectory

First, with the initial conditions set at (8, −19, 30), the system is integrated through 15 time steps. The time span is noted for the system in moving from the initial condition (8, −19, 30) along its trajectory to the attractor. This initial stage is termed the early time period (ETP) and denoted ${\rm{\Delta }}{t}_{ear\_s}^{({\rm{1}})}$. The superscript (1) refers to the first numerical test. The time interval spent by the trajectory in the left equilibrium-point region for the first time is denoted ${\rm{\Delta }}{t}_{r\_1}^{(1)}$ (here subscript *l_*1 refers to first residence in the left equilibrium-point region). Similarly, the time interval spent by the trajectory in the right equilibrium-point region for the first time is denoted as ${\rm{\Delta }}{t}_{r\_1}^{(1)}$ (by analogy, subscript *r_*1 refers to the first residence in the right equilibrium-point region). Analogous interpretations are given to ${\rm{\Delta }}{t}_{l\_{\rm{n}}}^{(1)}$ (*l_n* refers to the *n*-th time in the left region) and ${\rm{\Delta }}{t}_{r\_{\rm{n}}}^{(1)}$ (*r_n* refers to the *n*-th time in the right region). For the first numerical test, we obtain the following data: ${\rm{\Delta }}{t}_{ear\_s}^{(1)}={\rm{0.2}}$, ${\rm{\Delta }}{t}_{l\_1}^{(1)}={\rm{1.3}}$, ${\rm{\Delta }}{t}_{r\_1}^{(1)}={\rm{0.5}}$, ${\rm{\Delta }}{t}_{l\_{\rm{2}}}^{(1)}={\rm{1.8}}$, ${\rm{\Delta }}{t}_{r\_{\rm{2}}}^{(1)}={\rm{0.5}}$, ${\rm{\Delta }}{t}_{l\_{\rm{3}}}^{(1)}={\rm{1.8}}$, ${\rm{\Delta }}{t}_{r\_{\rm{3}}}^{(1)}={\rm{1.4}}$, ${\rm{\Delta }}{t}_{l\_{\rm{4}}}^{(1)}={\rm{1.7}}$, ${\rm{\Delta }}{t}_{r\_{\rm{4}}}^{(1)}={\rm{0.6}}$, ${\rm{\Delta }}{t}_{l\_{\rm{5}}}^{(1)}={\rm{1.6}}$, ${\rm{\Delta }}{t}_{r\_{\rm{5}}}^{(1)}={\rm{0.6}}$, ${\rm{\Delta }}{t}_{l\_{\rm{6}}}^{(1)}={\rm{0.9}}$, ${\rm{\Delta }}{t}_{r\_{\rm{6}}}^{(1)}={\rm{0.7}}$, ${\rm{\Delta }}{t}_{l\_{\rm{7}}}^{(1)}={\rm{1.4}}$.

Next, setting the initial conditions to (15, −14, 78), we performed the same procedure as above and again integrated the system through 15 time steps. The ETP is denoted ${\rm{\Delta }}{t}_{ear\_s}^{(2)}$ the time span for the first time the trajectory stays in its initial region and continue to obtain ${\rm{\Delta }}{t}_{l\_n}^{(2)}$ and ${\rm{\Delta }}{t}_{r\_n}^{(2)}$. Setting different initial conditions, we performed the test ten times in total. Table 1 presents a listing of all the experimental data.

**Table 1:** Residence times of the Lorenz trajectories moving to the left or right equilibrium point region.

No.	Initial conditions	ETP	Residence times of trajectories												
1	(8, −19, 30)	${\rm{\Delta }}{t}_{ear\_s}^{(1)}$	${\rm{\Delta }}{t}_{r\_1}^{(1)}$	${\rm{\Delta }}{t}_{l\_2}^{(1)}$	${\rm{\Delta }}{t}_{r\_2}^{(1)}$	${\rm{\Delta }}{t}_{l\_3}^{(1)}$	${\rm{\Delta }}{t}_{r\_3}^{(1)}$	${\rm{\Delta }}{t}_{l\_4}^{(1)}$	${\rm{\Delta }}{t}_{r\_4}^{(1)}$	${\rm{\Delta }}{t}_{l\_5}^{(1)}$	${\rm{\Delta }}{t}_{r\_5}^{(1)}$	${\rm{\Delta }}{t}_{l\_6}^{(1)}$	${\rm{\Delta }}{t}_{r\_6}^{(1)}$	${\rm{\Delta }}{t}_{l\_7}^{(1)}$	${\rm{\Delta }}{t}_{l\_1}^{(1)}$
		0.2	1.3	0.5	1.8	0.5	1.8	1.4	1.7	0.6	1.6	0.6	0.9	0.7	1.4
2	(15, −14, 78)	${\rm{\Delta }}{t}_{ear\_s}^{(2)}$	${\rm{\Delta }}{t}_{r\_1}^{({\rm{2}})}$	${\rm{\Delta }}{t}_{l\_1}^{({\rm{2}})}$	${\rm{\Delta }}{t}_{r\_{\rm{2}}}^{({\rm{2}})}$	${\rm{\Delta }}{t}_{l\_{\rm{2}}}^{({\rm{2}})}$	${\rm{\Delta }}{t}_{r\_{\rm{3}}}^{({\rm{2}})}$	${\rm{\Delta }}{t}_{l\_{\rm{3}}}^{({\rm{2}})}$	${\rm{\Delta }}{t}_{r\_{\rm{4}}}^{({\rm{2}})}$						
		0.66	2.69	1.0	1.65	4.1	1.6	1.7	1.6						
3	(14, −5, 90)	${\rm{\Delta }}{t}_{ear\_s}^{(3)}$	${\rm{\Delta }}{t}_{r\_1}^{({\rm{3}})}$	${\rm{\Delta }}{t}_{l\_1}^{({\rm{3}})}$	${\rm{\Delta }}{t}_{r\_{\rm{2}}}^{({\rm{3}})}$	${\rm{\Delta }}{t}_{l\_{\rm{2}}}^{({\rm{3}})}$	${\rm{\Delta }}{t}_{r\_{\rm{3}}}^{({\rm{3}})}$	${\rm{\Delta }}{t}_{l\_{\rm{3}}}^{({\rm{3}})}$	${\rm{\Delta }}{t}_{r\_{\rm{4}}}^{({\rm{3}})}$	${\rm{\Delta }}{t}_{l\_{\rm{4}}}^{({\rm{3}})}$					
		0.75	0.6	0.9	2.3	3.65	1.6	3.7	0.6	0.9					
4	(−12, 1, 8)	${\rm{\Delta }}{t}_{ear\_s}^{(4)}$	${\rm{\Delta }}{t}_{l\_1}^{({\rm{4}})}$	${\rm{\Delta }}{t}_{r\_1}^{({\rm{4}})}$	${\rm{\Delta }}{t}_{l\_2}^{({\rm{4}})}$	${\rm{\Delta }}{t}_{r\_2}^{({\rm{4}})}$	${\rm{\Delta }}{t}_{l\_3}^{({\rm{4}})}$	${\rm{\Delta }}{t}_{r\_3}^{({\rm{4}})}$	${\rm{\Delta }}{t}_{l\_4}^{({\rm{4}})}$	${\rm{\Delta }}{t}_{r\_4}^{({\rm{4}})}$					
		0.1	0.55	2.1	0.75	0.7	1.0	2.8	1.2	5.8					
5	(−5, 17, 20)	${\rm{\Delta }}{t}_{ear\_s}^{(5)}$	${\rm{\Delta }}{t}_{r\_1}^{({\rm{5}})}$	${\rm{\Delta }}{t}_{l\_1}^{({\rm{5}})}$	${\rm{\Delta }}{t}_{r\_{\rm{2}}}^{({\rm{5}})}$	${\rm{\Delta }}{t}_{l\_{\rm{2}}}^{({\rm{5}})}$	${\rm{\Delta }}{t}_{r\_{\rm{3}}}^{({\rm{5}})}$	${\rm{\Delta }}{t}_{l\_{\rm{3}}}^{({\rm{5}})}$	${\rm{\Delta }}{t}_{r\_{\rm{4}}}^{({\rm{5}})}$	${\rm{\Delta }}{t}_{l\_{\rm{4}}}^{({\rm{5}})}$	${\rm{\Delta }}{t}_{r\_{\rm{5}}}^{({\rm{5}})}$				
		0.2	0.3	1.0	1.5	3.0	0.5	1.0	1.5	3.2	2.8				
6	(8, −19, 30)	${\rm{\Delta }}{t}_{ear\_s}^{(6)}$	${\rm{\Delta }}{t}_{l\_1}^{({\rm{6}})}$	${\rm{\Delta }}{t}_{r\_1}^{({\rm{6}})}$	${\rm{\Delta }}{t}_{l\_2}^{({\rm{6}})}$	${\rm{\Delta }}{t}_{r\_2}^{({\rm{6}})}$	${\rm{\Delta }}{t}_{l\_3}^{({\rm{6}})}$	${\rm{\Delta }}{t}_{r\_3}^{({\rm{6}})}$	${\rm{\Delta }}{t}_{l\_4}^{({\rm{6}})}$	${\rm{\Delta }}{t}_{r\_4}^{({\rm{6}})}$	${\rm{\Delta }}{t}_{l\_{\rm{5}}}^{({\rm{6}})}$	${\rm{\Delta }}{t}_{r\_{\rm{5}}}^{({\rm{6}})}$			
		0.2	0.65	0.55	1.2	0.6	3.8	0.4	3.1	0.8	1.0	2.7			
7	(−16, 11, −40)	${\rm{\Delta }}{t}_{ear\_s}^{(7)}$	${\rm{\Delta }}{t}_{l\_1}^{({\rm{7}})}$	${\rm{\Delta }}{t}_{r\_1}^{({\rm{7}})}$	${\rm{\Delta }}{t}_{l\_2}^{({\rm{7}})}$	${\rm{\Delta }}{t}_{r\_2}^{({\rm{7}})}$	${\rm{\Delta }}{t}_{l\_3}^{({\rm{7}})}$	${\rm{\Delta }}{t}_{r\_3}^{({\rm{7}})}$	$\Delta {t}_{l\_4}^{({\rm{7}})}$	${\rm{\Delta }}{t}_{r\_4}^{({\rm{7}})}$	${\rm{\Delta }}{t}_{l\_{\rm{5}}}^{({\rm{7}})}$	${\rm{\Delta }}{t}_{r\_{\rm{5}}}^{({\rm{7}})}$	${\rm{\Delta }}{t}_{l\_{\rm{6}}}^{({\rm{7}})}$	${\rm{\Delta }}{t}_{r\_{\rm{6}}}^{({\rm{7}})}$	
		0.6	2.6	0.5	1.0	0.6	0.9	1.3	1.1	2.0	1.7	0.9	0.8	1.0	
8	(−11, −1, −50)	${\rm{\Delta }}{t}_{ear\_s}^{(8)}$	${\rm{\Delta }}{t}_{r\_1}^{({\rm{8}})}$	${\rm{\Delta }}{t}_{l\_1}^{({\rm{8}})}$	${\rm{\Delta }}{t}_{r\_{\rm{2}}}^{({\rm{8}})}$	${\rm{\Delta }}{t}_{l\_{\rm{2}}}^{({\rm{8}})}$	${\rm{\Delta }}{t}_{r\_{\rm{3}}}^{({\rm{8}})}$	${\rm{\Delta }}{t}_{l\_{\rm{3}}}^{({\rm{8}})}$	${\rm{\Delta }}{t}_{r\_{\rm{4}}}^{({\rm{8}})}$	${\rm{\Delta }}{t}_{l\_{\rm{4}}}^{({\rm{8}})}$	${\rm{\Delta }}{t}_{r\_{\rm{5}}}^{({\rm{8}})}$	${\rm{\Delta }}{t}_{l\_{\rm{5}}}^{({\rm{8}})}$	${\rm{\Delta }}{t}_{r\_{\rm{6}}}^{({\rm{8}})}$		
		0.9	0.6	1.0	2.2	2.3	0.6	1.8	1.4	1.7	0.5	1.0	1.0		
9	(15, 0, 55)	${\rm{\Delta }}{t}_{ear\_s}^{(9)}$	${\rm{\Delta }}{t}_{l\_1}^{({\rm{9}})}$	${\rm{\Delta }}{t}_{r\_1}^{({\rm{9}})}$	${\rm{\Delta }}{t}_{l\_2}^{({\rm{9}})}$	${\rm{\Delta }}{t}_{r\_2}^{({\rm{9}})}$	${\rm{\Delta }}{t}_{l\_3}^{({\rm{9}})}$								
		0.3	5.8	1.5	2.3	1.6	3.5								
10	(6, −19, −20)	${\rm{\Delta }}{t}_{ear\_s}^{(\mathrm{10})}$	${\rm{\Delta }}{t}_{l\_1}^{({\rm{10}})}$	${\rm{\Delta }}{t}_{r\_1}^{({\rm{10}})}$	${\rm{\Delta }}{t}_{l\_2}^{({\rm{10}})}$	${\rm{\Delta }}{t}_{r\_2}^{({\rm{10}})}$	${\rm{\Delta }}{t}_{l\_3}^{({\rm{10}})}$	${\rm{\Delta }}{t}_{r\_3}^{({\rm{10}})}$	${\rm{\Delta }}{t}_{l\_4}^{({\rm{10}})}$	${\rm{\Delta }}{t}_{r\_4}^{({\rm{10}})}$	${\rm{\Delta }}{t}_{l\_{\rm{5}}}^{({\rm{10}})}$				
		0.8	1.6	0.6	1.9	0.5	3.9	0.5	3.0	0.6	1.6				

**Figure 5:**
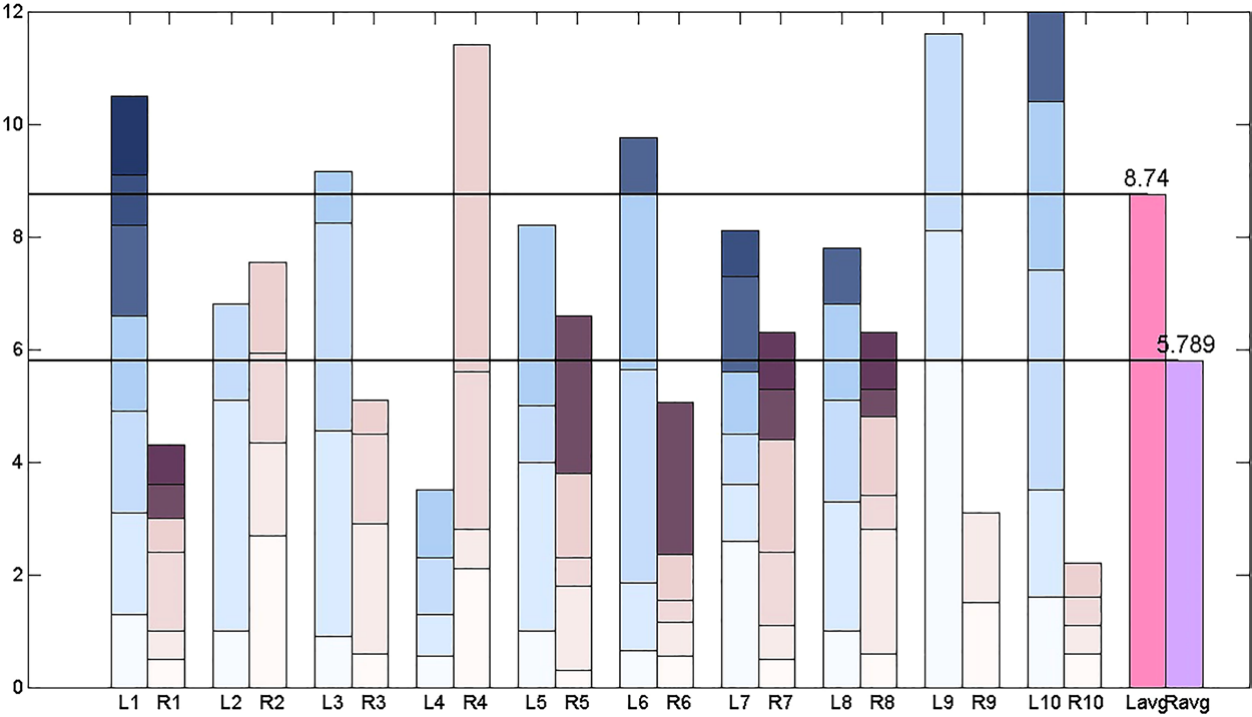
Residence time comparison chart of the Lorenz curve to the left or right equilibrium point region.

To compare the residence times of the trajectories in the left equilibrium-point region moving to the right region, a cumulative histogram of the residence times for all tests was constructed. In the histogram, *l*1 denotes the sum of the residence times of the first test in the left equilibrium region, specifically,

4a $$l{\rm{l}}=\sum _{i}^{7}{\rm{\Delta }}{t}_{l\_i}^{(1)},$$

whereas *r*1 denotes the sum of the residence times of the first test in the right equilibrium region, that is,

4b $$r{\rm{1}}=\sum _{i}^{6}{\rm{\Delta }}{t}_{r\_i}^{(1)};$$

similarly, for the remaining *ln* and *rn* (*n* = 2, .., 10). The quantities *lavg* and *ravg* are defined as

5a $$lavg=\frac{\sum _{k}^{10}lk}{{\rm{10}}},$$

5b $$ravg=\frac{\sum _{k}^{10}rk}{{\rm{10}}}$$

and give the average values of the residence times in the left and right equilibrium-point regions, respectively. From calculations, *lavg* = *8.74* whereas *ravg* = *5.789*. The difference in value means that the Lorenz trajectory favours the left equilibrium points locally and hence leads to a different conclusion from that in Section 3.2. The former is favoured globally whereas the latter is favoured locally. From the data (Table 1) and the cumulative histograms (Fig. 5), we find that the jumps in the Lorenz trajectory between left and right equilibrium points are irregular, and hence it is impossible to predict when the Lorenz trajectory is in the left or right equilibrium-point region. Concerning weather forecasting, a similar statement can be made; it is impossible to predict where the trajectory is. In actuality, sharp changes from drought to flood may take place, and such events may be caused by the chaotic nature of atmospheric flows.

## Conclusions and Outlook

Trajectory jumps of the Lorenz system from the stable region to the unstable region were studied, the main conclusions drawn being the following:

1. The stable and unstable regions of the Lorenz system are independent of the initial conditions and are inherent to the system;
2. The Lorenz trajectory favours in general different equilibrium points;
3. For different equilibrium point regions, the residence times of trajectories are also different.

The model on which weather forecasting is made is a nonautonomous system, equation (3), which is complex and nonstationary system. From view of mathematic principle, equation (3) is like (1), they should have the same feature, and for equation (3) there are also stable and unstable regions. The trajectory can favour different equilibrium point regions. The asymmetry arising from favoured regions may explain the occurrences of extreme events in the atmospheric flow system. Moreover, distinct residence times of trajectories in the different equilibrium point regions suggest that this may be a crucial aspect in the sharp change from one extreme to another such as drought and flooding. If the residence time of a trajectory for one equilibrium point region is longer, this state may then be the normal behaviour of the atmospheric system. In contrast if the time is shorter, this may be a rare instance in which an extreme event takes place^35^. A shorter residence time may be the dynamic attribute associated with such extreme events. In the study of extreme events in climate change, mainly statistical methods are used. Here, we provide a dynamic reason why this is so.

Different to previous studies^8–13,18–26^, using dynamics methods, the characteristics of stable and unstable regions of a dynamic system were the principle objectives from a theoretical and numerical test standpoint. The conclusion may explain the instability of the atmospheric system, and the method may provide a way to assess the abrupt changes seen in atmospheric observations.
